# 2-[(*E*)-(4-Fluoro­benzyl­imino)­meth­yl]-4-methyl­phenol

**DOI:** 10.1107/S1600536812027018

**Published:** 2012-06-20

**Authors:** Yue-Bao Jin, Ying Zhang, Yong-Kang Chang, Ke-Wei Lei

**Affiliations:** a State Key Lab. Base of Novel Functional Materials and Preparation Science, Institute of Solid Materials Chemistry, Faculty of Materials Science and Chemical Engineering, Ningbo University, Ningbo 315211, People’s Republic of China

## Abstract

In the title Schiff base compound, C_15_H_14_FNO, the benzene rings make a dihedral angle of 72.75 (13)°. The mol­ecular structure is stabilized by an intra­molecular O—H⋯N hydrogen bond. In the crystal, weak π–π stacking occurs between the phenol rings of inversion-related mol­ecules, the centroid–centroid distance being 3.7731 (14) Å.

## Related literature

For background and related compounds, see: Cohen *et al.* (1964 ▶); Xia *et al.* (2009 ▶).
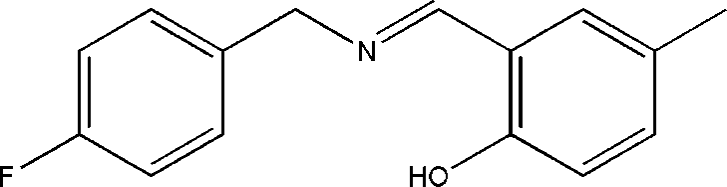


## Experimental

### 

#### Crystal data

C_15_H_14_FNO
*M* *_r_* = 243.28
Monoclinic, 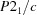

*a* = 15.0297 (9) Å
*b* = 6.1496 (3) Å
*c* = 14.3090 (9) Å
β = 104.142 (6)°
*V* = 1282.45 (13) Å^3^
*Z* = 4
Mo *K*α radiation
μ = 0.09 mm^−1^
*T* = 293 K
0.38 × 0.21 × 0.14 mm

#### Data collection

Rigaku R-AXIS RAPID diffractometer
9046 measured reflections
2265 independent reflections
1552 reflections with *I* > 2σ(*I*)
*R*_int_ = 0.030

#### Refinement

*R*[*F* ^2^ > 2σ(*F* ^2^)] = 0.060
*wR*(*F* ^2^) = 0.150
*S* = 1.10
2265 reflections
164 parameters
1 restraint
H-atom parameters constrained
Δρ_max_ = 0.15 e Å^−3^
Δρ_min_ = −0.17 e Å^−3^

### 

Data collection: *RAPID-AUTO* (Rigaku, 1998 ▶); cell refinement: *RAPID-AUTO*; data reduction: *CrystalStructure* (Rigaku/MSC, 2004 ▶); program(s) used to solve structure: *SHELXS97* (Sheldrick, 2008 ▶); program(s) used to refine structure: *SHELXL97* (Sheldrick, 2008 ▶); molecular graphics: *SHELXTL* (Sheldrick, 2008 ▶); software used to prepare material for publication: *SHELXL97*.

## Supplementary Material

Crystal structure: contains datablock(s) global, I. DOI: 10.1107/S1600536812027018/xu5548sup1.cif

Structure factors: contains datablock(s) 1. DOI: 10.1107/S1600536812027018/xu5548Isup2.hkl

Supplementary material file. DOI: 10.1107/S1600536812027018/xu5548Isup3.cml

Additional supplementary materials:  crystallographic information; 3D view; checkCIF report

## Figures and Tables

**Table 1:** Hydrogen-bond geometry (Å, °)

*D*—H⋯*A*	*D*—H	H⋯*A*	*D*⋯*A*	*D*—H⋯*A*
O1—H1⋯N1	0.82	1.90	2.628 (3)	147

**Fig. 1.**
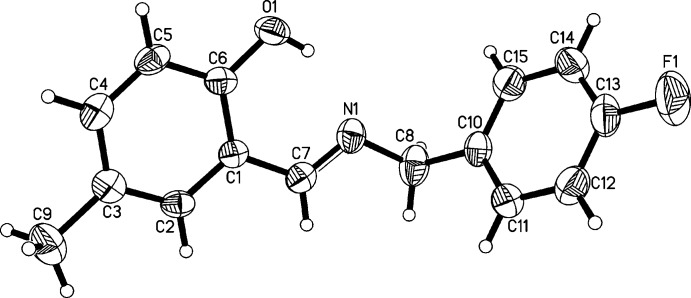
The structure of the title complex, showing 30% probability displacement ellipsoids and the atom-numbering scheme.

## supplementary crystallographic information

### Comment

Schiff base have played an important role in the development of coordination chemistry (Xia *et al.*, 2009) as they readily form stable complexes with most of the transition metals. Some of the reasons are that the N atom plays an important role in the formation of metal complexes, and that Schiff base compounds show photochromism and thermochromism in the solid state by proton transfer from the hydroxyl O atom to the imine N atom (Cohen *et al.*, 1964). Here we report on a new Schiff base.

The molecular structures of(I) illustrated in the Fig. 1. The C8 and N1 atoms form a 1.46 (4) Å single bond is longer than the double bond [1.28 (3) Å] formed by C7 and N1. The molecular structure is stabilized by an intramolecular O—H···N hydrogen bond.

### Experimental

2-Hydroxy-4-methylbenzaldehyde (20 mmol, 2.72 g) and (4-fluorophenyl)methanamine (20 mmol, 2.5 g) were dissolved in ethanol respectively. Then put them together and the solution was refluxed for 30 min. Yellow powder precipitates when cooled to room temperature. After evaporation, a crude product was recrystallized twice from methanol to give yellow crystals.

### Refinement

H atoms were placed in geometrically idealized positions and constrained to ride on their parent atoms (C—H = 0.93– 0.97, O—H = 0.82 Å). *U*_iso_(H) = 1.5*U*_eq_(O) and 1.2*U*_eq_(C).

### Crystal data

**Table:**

C_15_H_14_FNO	*F*(000) = 512.0
*M**_r_* = 243.28	*D*_x_ = 1.260 Mg m^−^^3^
Monoclinic, *P*2_1_/*c*	Mo *K*α radiation, λ = 0.71073 Å
Hall symbol: -P 2ybc	Cell parameters from 2338 reflections
*a* = 15.0297 (9) Å	θ = 1.0–25.0°
*b* = 6.1496 (3) Å	µ = 0.09 mm^−^^1^
*c* = 14.3090 (9) Å	*T* = 293 K
β = 104.142 (6)°	Block, yellow
*V* = 1282.45 (13) Å^3^	0.38 × 0.21 × 0.14 mm
*Z* = 4	

### Data collection

**Table:**

Rigaku R-AXIS RAPID diffractometer	1552 reflections with *I* > 2σ(*I*)
Radiation source: fine-focus sealed tube	*R*_int_ = 0.030
Graphite monochromator	θ_max_ = 25.0°, θ_min_ = 2.8°
ω scans	*h* = −17→17
9046 measured reflections	*k* = −7→7
2265 independent reflections	*l* = −16→17

### Refinement

**Table:**

Refinement on *F*^2^	Primary atom site location: structure-invariant direct methods
Least-squares matrix: full	Secondary atom site location: difference Fourier map
*R*[*F*^2^ > 2σ(*F*^2^)] = 0.060	Hydrogen site location: inferred from neighbouring sites
*wR*(*F*^2^) = 0.150	H-atom parameters constrained
*S* = 1.10	*w* = 1/[σ^2^(*F*_o_^2^) + (0.0477*P*)^2^ + 0.4777*P*] where *P* = (*F*_o_^2^ + 2*F*_c_^2^)/3
2265 reflections	(Δ/σ)_max_ < 0.001
164 parameters	Δρ_max_ = 0.15 e Å^−^^3^
1 restraint	Δρ_min_ = −0.17 e Å^−^^3^

### Special details

**Table:**

Geometry. All e.s.d.'s (except the e.s.d. in the dihedral angle between two l.s. planes) are estimated using the full covariance matrix. The cell e.s.d.'s are taken into account individually in the estimation of e.s.d.'s in distances, angles and torsion angles; correlations between e.s.d.'s in cell parameters are only used when they are defined by crystal symmetry. An approximate (isotropic) treatment of cell e.s.d.'s is used for estimating e.s.d.'s involving l.s. planes.
Refinement. Refinement of *F*^2^ against ALL reflections. The weighted *R*-factor *wR* and goodness of fit *S* are based on *F*^2^, conventional *R*-factors *R* are based on *F*, with *F* set to zero for negative *F*^2^. The threshold expression of *F*^2^ > σ(*F*^2^) is used only for calculating *R*-factors(gt) *etc*. and is not relevant to the choice of reflections for refinement. *R*-factors based on *F*^2^ are statistically about twice as large as those based on *F*, and *R*- factors based on ALL data will be even larger.

### Fractional atomic coordinates and isotropic or equivalent isotropic displacement parameters (Å^2^)

**Table:**

	*x*	*y*	*z*	*U*_iso_*/*U*_eq_	
C1	0.02053 (15)	0.5287 (4)	0.65069 (15)	0.0460 (6)	
O1	0.10373 (12)	0.2040 (3)	0.63178 (13)	0.0702 (5)	
H1	0.1454	0.2793	0.6633	0.105*	
C7	0.10205 (17)	0.6427 (4)	0.70213 (16)	0.0553 (6)	
H7A	0.0966	0.7852	0.7218	0.066*	
C6	0.02389 (17)	0.3154 (4)	0.61582 (16)	0.0515 (6)	
C5	−0.05629 (19)	0.2196 (4)	0.56346 (18)	0.0606 (7)	
H5A	−0.0550	0.0787	0.5402	0.073*	
C3	−0.14383 (18)	0.5419 (4)	0.57872 (17)	0.0587 (7)	
C2	−0.06444 (16)	0.6353 (4)	0.63143 (16)	0.0533 (6)	
H2A	−0.0671	0.7752	0.6553	0.064*	
N1	0.18143 (15)	0.5538 (4)	0.72148 (15)	0.0637 (6)	
C4	−0.13757 (18)	0.3317 (5)	0.54576 (17)	0.0624 (7)	
H4A	−0.1905	0.2642	0.5103	0.075*	
C9	−0.23392 (19)	0.6640 (6)	0.5563 (2)	0.0902 (10)	
H9A	−0.2224	0.8168	0.5521	0.135*	
H9B	−0.2649	0.6388	0.6066	0.135*	
H9C	−0.2717	0.6140	0.4960	0.135*	
C10	0.32939 (16)	0.7009 (5)	0.71083 (18)	0.0594 (7)	
C8	0.25963 (19)	0.6878 (6)	0.7706 (2)	0.0798 (9)	
H8A	0.2878	0.6250	0.8330	0.096*	
H8B	0.2385	0.8328	0.7809	0.096*	
F1	0.51784 (14)	0.7389 (4)	0.54697 (17)	0.1337 (9)	
C15	0.38998 (19)	0.5337 (5)	0.7092 (2)	0.0754 (8)	
H15A	0.3883	0.4105	0.7464	0.090*	
C13	0.45416 (19)	0.7270 (6)	0.5999 (2)	0.0800 (9)	
C11	0.33333 (18)	0.8798 (5)	0.6546 (2)	0.0701 (8)	
H11A	0.2928	0.9943	0.6544	0.084*	
C14	0.45313 (19)	0.5445 (6)	0.6536 (2)	0.0840 (9)	
H14A	0.4937	0.4308	0.6529	0.101*	
C12	0.3958 (2)	0.8944 (5)	0.5983 (2)	0.0795 (9)	
H12A	0.3976	1.0163	0.5604	0.095*	

### Atomic displacement parameters (Å^2^)

**Table:**

	*U*^11^	*U*^22^	*U*^33^	*U*^12^	*U*^13^	*U*^23^
C1	0.0579 (14)	0.0454 (13)	0.0398 (12)	−0.0019 (11)	0.0216 (10)	0.0035 (10)
O1	0.0752 (12)	0.0557 (11)	0.0887 (13)	0.0113 (9)	0.0373 (10)	−0.0010 (10)
C7	0.0664 (16)	0.0565 (15)	0.0483 (13)	−0.0059 (12)	0.0244 (12)	−0.0014 (12)
C6	0.0675 (16)	0.0451 (14)	0.0507 (13)	0.0023 (12)	0.0312 (12)	0.0029 (11)
C5	0.0812 (18)	0.0508 (15)	0.0591 (15)	−0.0124 (14)	0.0347 (14)	−0.0112 (13)
C3	0.0670 (16)	0.0643 (17)	0.0475 (13)	0.0028 (13)	0.0191 (12)	0.0075 (13)
C2	0.0694 (16)	0.0446 (13)	0.0497 (13)	0.0042 (12)	0.0219 (12)	0.0033 (11)
N1	0.0596 (13)	0.0755 (15)	0.0597 (13)	−0.0104 (12)	0.0216 (11)	−0.0014 (11)
C4	0.0697 (17)	0.0713 (18)	0.0494 (14)	−0.0164 (14)	0.0207 (13)	−0.0049 (14)
C9	0.0684 (19)	0.105 (3)	0.092 (2)	0.0169 (17)	0.0095 (17)	0.018 (2)
C10	0.0463 (14)	0.0720 (18)	0.0561 (15)	−0.0083 (13)	0.0050 (11)	−0.0012 (14)
C8	0.0684 (18)	0.110 (2)	0.0631 (17)	−0.0216 (17)	0.0198 (14)	−0.0138 (17)
F1	0.1096 (15)	0.160 (2)	0.159 (2)	−0.0252 (14)	0.0876 (15)	−0.0185 (16)
C15	0.0673 (18)	0.074 (2)	0.0802 (19)	−0.0018 (15)	0.0096 (15)	0.0124 (16)
C13	0.0612 (18)	0.097 (3)	0.089 (2)	−0.0158 (17)	0.0329 (16)	−0.009 (2)
C11	0.0580 (16)	0.0698 (19)	0.0807 (19)	0.0033 (14)	0.0136 (14)	−0.0013 (16)
C14	0.0587 (17)	0.083 (2)	0.109 (3)	0.0091 (16)	0.0190 (17)	−0.012 (2)
C12	0.078 (2)	0.075 (2)	0.087 (2)	−0.0110 (17)	0.0240 (17)	0.0117 (17)

### Geometric parameters (Å, º)

**Table:**

C1—C2	1.402 (3)	C9—H9B	0.9600
C1—C6	1.409 (3)	C9—H9C	0.9600
C1—C7	1.447 (3)	C10—C11	1.373 (4)
O1—C6	1.352 (3)	C10—C15	1.378 (3)
O1—H1	0.8200	C10—C8	1.508 (3)
C7—N1	1.280 (3)	C8—H8A	0.9700
C7—H7A	0.9300	C8—H8B	0.9700
C6—C5	1.385 (3)	F1—C13	1.360 (3)
C5—C4	1.371 (4)	C15—C14	1.381 (4)
C5—H5A	0.9300	C15—H15A	0.9300
C3—C2	1.372 (3)	C13—C12	1.349 (4)
C3—C4	1.387 (4)	C13—C14	1.362 (4)
C3—C9	1.513 (4)	C11—C12	1.381 (4)
C2—H2A	0.9300	C11—H11A	0.9300
N1—C8	1.466 (3)	C14—H14A	0.9300
C4—H4A	0.9300	C12—H12A	0.9300
C9—H9A	0.9600		
			
C2—C1—C6	118.4 (2)	H9A—C9—H9C	109.5
C2—C1—C7	119.4 (2)	H9B—C9—H9C	109.5
C6—C1—C7	122.2 (2)	C11—C10—C15	117.7 (3)
C6—O1—H1	109.5	C11—C10—C8	120.7 (3)
N1—C7—C1	122.1 (2)	C15—C10—C8	121.6 (3)
N1—C7—H7A	118.9	N1—C8—C10	110.2 (2)
C1—C7—H7A	118.9	N1—C8—H8A	109.6
O1—C6—C5	119.7 (2)	C10—C8—H8A	109.6
O1—C6—C1	121.3 (2)	N1—C8—H8B	109.6
C5—C6—C1	119.1 (2)	C10—C8—H8B	109.6
C4—C5—C6	120.3 (2)	H8A—C8—H8B	108.1
C4—C5—H5A	119.9	C10—C15—C14	121.6 (3)
C6—C5—H5A	119.9	C10—C15—H15A	119.2
C2—C3—C4	117.1 (2)	C14—C15—H15A	119.2
C2—C3—C9	121.4 (3)	C12—C13—F1	119.6 (3)
C4—C3—C9	121.5 (3)	C12—C13—C14	122.7 (3)
C3—C2—C1	122.7 (2)	F1—C13—C14	117.7 (3)
C3—C2—H2A	118.7	C10—C11—C12	121.8 (3)
C1—C2—H2A	118.7	C10—C11—H11A	119.1
C7—N1—C8	117.3 (3)	C12—C11—H11A	119.1
C5—C4—C3	122.5 (2)	C13—C14—C15	118.0 (3)
C5—C4—H4A	118.8	C13—C14—H14A	121.0
C3—C4—H4A	118.8	C15—C14—H14A	121.0
C3—C9—H9A	109.5	C13—C12—C11	118.2 (3)
C3—C9—H9B	109.5	C13—C12—H12A	120.9
H9A—C9—H9B	109.5	C11—C12—H12A	120.9
C3—C9—H9C	109.5		

### Hydrogen-bond geometry (Å, º)

**Table:**

*D*—H···*A*	*D*—H	H···*A*	*D*···*A*	*D*—H···*A*
O1—H1···N1	0.82	1.90	2.628 (3)	147
